# Case Presentation of Duodenal Obstruction Caused by Complete Annular Pancreas

**DOI:** 10.5334/jbsr.3432

**Published:** 2024-01-11

**Authors:** Ahmet Bozer, Zehra Hilal Adıbelli

**Affiliations:** 1 Department of Radiology, Bozyaka Education and Research Hospıtal, İzmir, Turkey; 2 Department of Radiology, Bozyaka Education and Research Hospıtal, İzmir, Turkey

**Keywords:** Duodenal obstruction, complete annular pancreas, recurrent vomiting, congenital anomaly

## Abstract

**Teaching Point:** A complete annular pancreas, a rare congenital anomaly, can lead to duodenal obstruction, causing recurrent symptoms like vomiting and often requiring surgical intervention for relief.

A 43-year-old male patient presented with recurrent vomiting episodes for the past 2 weeks. The patient reported a history of unexplained vomiting episodes over the past 10 years. Laboratory tests revealed borderline elevated levels of liver function tests, amylase, and lipase. Abdominal ultrasonography yielded normal results. However, a computed tomography (CT) scan showed that the second duodenum was surrounded by pancreatic tissue (Figure 1A and B, arrowhead), resulting in proximal dilation of the duodenum and the stomach (Figure 1B, arrow). The pancreas tissue encircling the duodenum completely was also demonstrated by magnetic resonance imaging (MRI) (Figure 1C and D, arrowhead). This constellation of findings is indicative of a congenital pancreatic anomaly termed ‘Complete Annular Pancreas’. It is crucial to distinguish the complete annular pancreas from the incomplete annular pancreas, which does not fully encircle the second duodenum.

**Figure 1:**
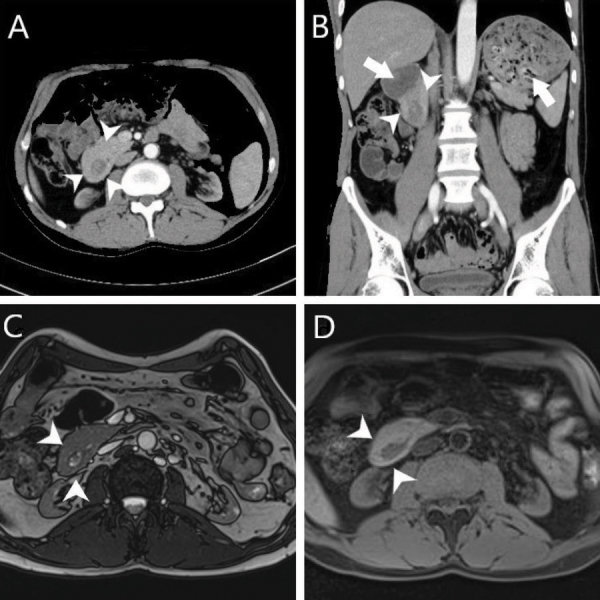
A case of the complete annular pancreas causing duodenal obstruction.

Complete annular pancreas is an exceptionally uncommon anomaly, manifesting in one out of 20,000 people. This developmental anomaly occurs in the fifth week of embryogenesis when two segments of a ventral pancreatic bud undergo migration in opposite directions, thus encircling the duodenum. This event might be a consequence of abnormal hedgehog signaling, the pathway that transmits information to embryonic cells for proper differentiation and migration. Severe duodenal obstruction is typically observed in the neonatal period, but it can also manifest as recurring vomiting in individuals aged 20 to 50 years. Various approaches, such as gastroscopy, CT scans, MRI, endoscopic ultrasound, or laparotomy, can determine the diagnosis.

In cases of symptomatic complete annular pancreas, surgical intervention is the preferred treatment. Bypass surgeries such as duodeno-jejunostomy or gastro-jejunostomy are the primary surgical approaches employed to alleviate the obstruction and provide symptomatic relief [1].

## Declarations

Ethics approval and consent to participate.

The procedure followed institutional ethics and complied with the WMA Declaration of Helsinki for medical research involving human subjects.
